# Brief Review: Ergospirometry in Patients with Obstructive Sleep Apnea Syndrome

**DOI:** 10.3390/jcm7080191

**Published:** 2018-07-31

**Authors:** Vasileios Stavrou, Fotini Bardaka, Eleni Karetsi, Zoe Daniil, Konstantinos I. Gourgoulianis

**Affiliations:** Laboratory of Cardio-Pulmonary Testing, Department of Respiratory Medicine, University of Thessaly, 41110 Larissa, Greece; bardakafotini@yahoo.gr (F.B.); ekaretsi@med.uth.gr (E.K.); zdaniil@med.uth.gr (Z.D.); kgourg@med.uth.gr (K.I.G.)

**Keywords:** obstructive sleep apnea, exercise, maximal oxygen uptake

## Abstract

This brief review summarizes the available literature on the intersection of obstructive sleep apnea syndrome (OSAS) and ergospirometry. Ergospirometry provides an assessment of integrative exercise responses involving pulmonary, cardiovascular, neuropsychological, and skeletal muscle systems, which are not adequately reflected through the measurement of individual organ system functions. Sleep disorders, including OSAS, often exacerbate problems in the operation of the autonomic nervous system, heart function, lung mechanics, anxiety, and muscle metabolism. Patients with OSAS have low aerobic capacity due to dysfunction of these systems, which often affect quality of sleep. Further research is necessary to elucidate the precise mechanisms through which ergospirometry can be useful in the assessment and early identification of patients with OSAS.

## 1. Ergospirometry

Ergospirometry (cardiopulmonary exercise testing) according to Albouaini et al. [1] provides an assessment of integrative exercise responses involving pulmonary, cardiovascular, neuropsychological, and skeletal muscle systems, which are not adequately reflected through the measurement of individual organ system functions. This non-invasive, dynamic physiological overview allows for the evaluation of both submaximal and overdosed responses, providing the health professional with relevant information for clinical decision making. Ergospirometry is increasingly used in a wide range of clinical applications for the evaluation of undiagnosed exercise intolerance and for the objective determination of functional capacity and impairment to aerobic ability. Its use in patient management suggests that resting pulmonary and cardiac function testing cannot reliably predict exercise performance and functional capacity, and that overall health status correlates better with exercise tolerance than with resting measurements [1]. Ergospirometry involves measurements of respiratory oxygen uptake (*V*O_2_), carbon dioxide production (*V*CO_2_), and ventilation during a symptom-limited exercise test [2].

## 2. Obstructive Sleep Apnea Syndrome

Obstructive sleep apnea syndrome (OSAS) is characterized by recurrent upper airway collapse during sleep, leading to intermittent nocturnal hypoxia and sleep fragmentation. OSAS symptomatology appears as a reduction (hypopnea) or complete cessation (apnea) of airflow through the airways despite continued respiratory efforts. OSAS is diagnosed by clinical history and polysomnography (PSG). OSAS is classified by an apnea-hypopnea index (AHI > 15 or an AHI > five with daytime and nighttime symptoms. The apnea severity is classified as mild (AHI five to 15), moderate (AHI 15.01 to 30), or severe (AHI > 30.1) [3].

## 3. Exercise in OSAS Patients

**Figure 1:**
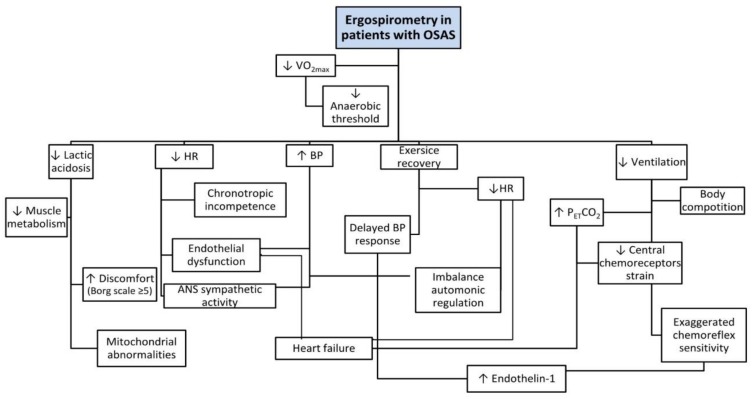
Cardiopulmonary responses to exercise in OSAS.

Compared to the general population, OSAS patients exhibit lower aerobic (*V*O_2max_) and anaerobic threshold (AT) capacity [4,5,6,7]. This is attributed to the pathophysiology of OSAS in patients, including lower sleep quality that causes disturbed exercise responses [6,8] (see Table 1). Low quality of sleep has a negative effect on exercise (Figure 1) for both OSAS patients and healthy people. According to Chennaoui et al. [9] the performance in exercise depends on physiological, psychological, and biomechanical parameters. There are conflicting studies in the correlation between performance and sleep loss: Chen et al. [10] suggested that poor sleep quality reduces *V*O_2max_ while Hill et al. [11] found no significant differences in *V*O_2max_ and loss of sleep. OSAS patients showed reduced exercise capacity (↓*V*O_2max_), and a decreased response of HR to exercise compared to normal people, which was attributed to chronotropic incompetence [5]. In addition, OSAS patients experienced increased systolic and diastolic blood pressure (↑BP) during exercise and permanently high systolic blood pressure (>200 mmHg) during the first minutes of post-exercise recovery. According to Aron et al. [5] these differences may be due to cardiac dysfunction, decreased muscle metabolism, chronic hyper-activation of the sympathetic nervous system, and endothelial dysfunction [9].

## 4. Responses in OSAS Patients during Exercise

### 4.1. Autonomic Nervous System and Heart Dysfunction

The recurrent hypoxia-re-oxygenation that characterizes OSAS results in instability of the autonomic nervous system (ANS) [24], which is associated with endothelial dysfunction, sympathetic induced vasoconstriction, and increased beta 2 receptor response [8,25,26]. According to Mansukhani et al. [24] there are various mechanisms through which blood pressure is altered during exercise in patients with OSAS, such as impaired vagal activity, increased platelet aggregation, and/or insulin resistance that may impair left ventricular function [27].

According to Somers et al. [28], hypoxia is a potent stimulator for the sympathetic nervous system. Fluctuations in sympathetic and parasympathetic activity in patients with OSAS may lead to the development of atrial and ventricular arrhythmias [29]. During rapid eyes movement (REM) sleep, OSAS patients have suspended central homeostasis, allowing large fluctuations in respiration, thermoregulation, and circulation. Moreover, the activity of the sympathetic nervous system and hemodynamic alterations have an impact on blood pressure and heart rate, leading to higher values compared to those of normal individuals [26]. These alterations are often associated with the instant restoration of muscle tone during REM sleep, leading to abrupt disruption of the sympathetic nervous system and increased blood pressure [8]. Furthermore, the restoration of muscle tone possibly relates to increased blood pressure, according to baroreceptor-reflex mediated inhibition of sympathetic activity [26] and an adaptation of the baroreceptor-reflex during sleep, as reflected by the intense tachycardia and sympathetic stimulation with comparatively mild hypotension at the point of awakening [26,30].

### 4.2. Lung Mechanics and Gas Exchange

On the other hand, reduced pulmonary ventilation activity (↓V_E_) is associated with the frequency of apnea and desaturation during sleep, interpreting an increased airway resistance during sleep [23,31]. Abdeyrim et al. [32] observed that FRC and ERV decreased in obese OSAS patients regardless of body mass index whereas these reductions had a negative correlation with the severity of OSAS. They also found that OSAS had a negative effect on pulmonary tumors and may be due to the abnormally elevated recoil pressure of lung elasticity in patients with sleep apnea syndrome.

Patients with OSAS due to repeated hypoxia-reoxygenation can exhibit carbon dioxide (CO_2_) retention and therefore respiratory acidosis, resulting in compensatory renal retention of bicarbonate ions and excretion of hydrogen ions. The elevated bicarbonate levels may cause a respiratory center response, resulting in reduced respiratory frequency, and also metabolic alkalosis [33], inducing compensatory respiratory acidosis through reduced daily ventilation. In addition, if the low breathing frequency is insufficient to eliminate extra CO_2_ produced during exercise, it can cause increased levels of partial arterial pressure of CO_2_ (PaCO_2_) and end-tidal CO_2_ pressure (P_ET_CO_2_) [34]. The increased P_ET_CO_2_ may be an end product of a complex conglomerate, influenced by factors, such as the severity of sleep apnea, daytime partial arterial pressure of oxygen (PaO_2_), blunted respiratory drive, respiratory mechanics, and respiratory muscle fatigue [35].

### 4.3. Anxiety

Several studies have adequately explained the relationship between sleep loss and performance in ergospirometry testing while few studies have dealt with psychological effects [36]. According to Matsumoto et al. [37], the combination of sleep loss and exercise have a negative impact on cognitive performance, such as increased cognitive anxiety, anxiety for failure, memory impairment, reduced concentration, and dysfunctional affective regulation that affects physical performance in endurance sports [38].

### 4.4. Muscle Metabolism

An important factor, which restricts ergospirometry testing on OSAS patients, is related to their metabolic profile; it has been observed that low *V*O_2peak_ is associated with premature fatigue of the lower limbs [18]. The premature fatigue of the lower limbs by the 1–10 Borg Scale (Borg scale ≥5) is associated with decreased muscle metabolism during ergospirometry due to long-term exposure to hypoxemia and subsequent adaptations to affect muscle tissue [13,39,40]. Muscle weakness and leg effort are different sensations, but are recorded as leg fatigue and may limit effort in ergospirometry, [41] while the intensity of the leg pain symptoms is very subjective [15]. The average person can stand a higher degree of fatigue (very severe fatigue, ≥6 Borg Scale) compared to most OSAS patients, who can stand moderate fatigue, resulting in premature discontinuation of exercise [9]. This disorder of muscle metabolism is associated with elevated levels of lactic acid concentration in the blood and decreased ability to remove it in patients with sleep disorders during exercise [7].

Previous studies have shown decreased exercise capacity in patients with OSAS due to exercise intolerance and tenderness in leg fatigue [42]. These symptoms are associated with the attenuation of oxidative metabolism, which can be explained by the occurrence of mitochondrial abnormalities in muscle fibers, similar to the phenomenon observed in normal people when exposed to chronic altitude hypoxia and could possibly explain the increased production of reactive species of oxygen present in neutrophils of OSAS patients [13]. According to Vanuxen et al. [7], leg fatigue is a metabolic muscle injury and the change in lactic acid concentration during exercise has been proven during the peak testing. In addition, delayed lactic acid removal has been observed due to disturbance of oxidoglycololytic metabolism [22] and changes in skeletal muscle fibers [40] due to muscle adaptation to chronic hypoxia [13].

## 5. Conclusions

This review reveals reduced ability of patients with OSAS in aerobic capacity due to dysfunction between cardiopulmonary, neurological and skeletal muscle systems which relate to quality of sleep. The reduced maximal aerobic capacity is associated with increased cardiovascular risks and ergospirometry can be useful in the assessment and early identification of patients with OSAS.

## Figures and Tables

**Table 1:** Presenting research results in patients with OSAS during ergospirometry.

Reference	Subjects	Exercise-Protocols	Duration	Results
Grote et al., 2004 [8]	1149	50 W/2-min^−1^	Acute	↑BP, ↓HR
Tryfon et al., 2004 [12]	27	10, 15 or 20 W/1-min^−1^	Acute	↓*V*O_2max_, ↑BP
Bonnanietal., 2004 [13]	20	3-min sub-maximal	Acute	↓*V*O_2max_,↓La
Oztruk et al., 2005 [14]	30	20 W/2-min^−1^	Acute	↓*V*O_2max_
Linetal., 2006 [15]	40	1 min on 100 Kpm	Acute	↓*V*O_2max_, ↓AT, cardiac dysfunction
Kaleth et al., 2007 [16]	32	15 W/1-min^−1^	Acute	↓*V*O_2max_, ↓HR, ↓SBP
Vanhecke et al., 2008 [17]	92	Bruce treadmill	Acute	↓*V*O_2max_, ↓HR, ↑BP
Ucok et al., 2009 [18]	40	Astrand and Wingate test	Acute	↓*V*O_2max_, ↑ % body fat
Nanasetal., 2010 [19]	21	Incremental CPET on treadmill	Acute	↓*V*O_2max_, ↓HR recovery, ↓ANS
Cintraetal., 2011 [4]	261	Maximal test	Acute	↓HDL, ↑LV, ↑BP
Ackel-D’Eliaetal., 2012 [20]	22	Pulmonary Rehabilitation	3 days per week, 1 h per day, 2 months	↑ESS, ↑quality of life
Rizzi et al., 2013 [21]	31	10-15 W/1-min^−1^	Acute	↓*V*O_2max_, ↑DBP
Beitler et al., 2014 [22]	15	10-15 W/1-min^−1^	Acute	↓*V*O_2max_
Stavrou et al., 2015 [23]	21	15-20 W/1-min^−1^	Acute	↓*V*O_2max_, ↓V_E_/MVV, ↓O_2pulse_

**Abbreviations:** ANS: autonomic nervous activity; AT: anaerobic threshold; BP: blood pressure; CPET: cardiopulmonary exercise testing; DBP: diastolic blood pressure; ESS: Epworth sleep scale; HDL: high density lipoprotein; HR: heart rate; La: lactic acidosis; LV: left ventricular; MVV: maximal voluntary volume; O_2pulse_: *V*O_2_/HR; SBP: systolic blood pressure; V_E_: ventilation; *V*O_2max_: maximal oxygen uptake; W: watts.

## References

[1] Albouaini K., Egred M., Alahmar A. (2007). Cardiopulmonary exercise testing and its application. Postgrad. Med. J..

[2] Wasserman K., Hansen J.E., Sue D.Y., Stringer W.W., Whipp B. (2004). Principles of Exercise Testing and Interpretation: Including Pathophysiology and Clinical Applications.

[3] Mendes F.A., Marone S.M., Duarte B.B., Arenas A.P. (2014). Epidemiologic profile of patients with snoring and obstructive sleep apnea in a University Hospital. Int. Arch. Otorhinolaryngol..

[4] Cintra F.D., Tufik S., Paola A., Feres M.C., Melo-Fujita L., Oliveira W., Rizzi C., Poyares D. (2011). Cardiovascular profile in patients with obstructive sleep apnea. Arquivos Bras. Cardiol..

[5] Aron A., Zedalis D., Gregg J.M., Gwazdauskas F.C., Herbert W.G. (2009). Potential clinical use of cardiopulmonary exercise testing in obstructive sleep apnea hypopnea syndrome. Int. J. Cardiol..

[6] Guillermo L.Q., Gal T.J., Mair E.A. (2006). Does obstructive sleep apnea affect aerobic fitness?. Ann. Otol. Rhinol. Laryngol..

[7] Vanuxem D., Badier M., Guillot C., Delpierre S., Jahjah F., Vanuxem P. (1997). Impairment of muscle energy metabolism in patients with sleep apnoea syndrome. Respir. Med..

[8] Grote L., Hedner J., Peter H. (2004). The heart rate response to exercise is blunted in patients with sleep-related breathing disorder. Cardiology.

[9] Chennaoui M., Arnal P.J., Sauvet F., Leger D. (2015). Sleep and exercise: A reciprocal issue?. Sleep Med. Rev..

[10] Chen H.I. (1991). Effects of 30-h sleep loss on cardiorespiratory functions at rest and in exercise. Med. Sci. Sports Exerc..

[11] Hill D.W., Borden D.O., Darnaby K.M., Hendricks D.N. (1994). Aerobic and anaerobic contributions to exhaustive high-intensity exercise after sleep deprivation. J. Sports Sci..

[12] Tryfon S., Stanopoulos I., Dascalopoulou E., Argyropoulou P., Bouros D., Mavrofridis E. (2004). Sleep apnea syndrome and diastolic blood pressure elevationduring exercise. Respiration.

[13] Bonanni E., Pasquali L., Manca M.L., Maestri M., Prontera C., Fabbrini M., Berrettini S., Zucchelli G., Siciliano G., Murri L. (2004). Lactate production and catecholamine profile during aerobic exercise in normotensive OSAS patients. Sleep Med..

[14] Ozturk L.M., Metin G., Cuhadaroglu C., Utkusavas A., Tutluoglu B. (2005). Cardiopulmonary responses to exercise in moderate-to-severe obstructive sleep apnea. Tuberk Toraks.

[15] Lin C.C., Hsieh W.Y., Chou C.S., Liaw S.F. (2006). Cardiopulmonary exercise testing in obstructive sleep apnea syndrome. Respir. Physiol. Neurobiol..

[16] Kaleth A.S., Chittenden T.W., Hawkins B.J., Hargens T.A., Guill S.G., Zedalis D., Gregg J.M., Herbert W.G. (2007). Unique cardiopulmonary exercise test responses in overweight middle-aged adults with obstructive sleep apnea. Sleep Med..

[17] Vanhecke T., Franklin B., Zalesin K., Sangal B., deJong A., Agrawal V., McCullough P. (2008). Cardiorespiratory fitness and obstructive sleep apnea syndrome in morbidly obese patients. Chest.

[18] Ucok K., Aycicek A., Sezer M., Genc A., Akkaya M., Caglar V., Fidan F., Unlu M. (2009). Aerobic and anaerobic exercise capacities in obstructive sleep apnea and associations with subcutaneous fat distributions. Lung.

[19] Nanas S., Sakellariou D., Kapsimalakou S., Dimopoulos S., Tassiou A., Tasoulis A., Anastasiou-Nana M., Vagiakis E., Roussos C. (2010). Heart rate recovery and oxygen kinetics after exercise in obstructive sleep apnea syndrome. Clin. Cardiol..

[20] Ackel-D’Elia C., da Silva A.C., Silva R.S., Truksinas E., Sousa B.S., de Mello M.T., Bittencourt L.R. (2012). Effects of exercise training associated with continuous positive airway pressure treatment in patients with obstructive sleep apnea syndrome. Sleep Breath..

[21] Rizzi C.F., Cintra F., Mello-Fujita L., Rios L.F., Mendonca E.T., Feres M.C., Poyares D. (2013). Does obstructive sleep apnea impair the cardiopulmonary response to exercise?. Sleep.

[22] Beitler J.R., Awad K.M., Bakker J.P., Edwards B.A., DeYoung P., Djonlagic I., Malhotra A. (2014). Obstructive Sleep Apnea Is Associated with Impaired Exercise Capacity: A Cross-Sectional Study. J. Clin. Sleep Med..

[23] Stavrou V., Vavougios G., Pastaka C., Daniil Z., Gourgoulianis K., Karetsi E. The cardiopulmonary exercise testing as a novel predictive tool of sleep apnea syndrome. Proceedings of the ERS International Congress.

[24] Mansukhani M.P., Allison T.G., Lopez-Jimenez F., Somers V.K., Caples S.M. (2013). Functional aerobic capacity in patients with sleep-disordered breathing. Am. J. Cardiol..

[25] Carlson J.T., Hedner J.A., Sellgren J., Elam M., Wallin B.G. (1996). Depressed baroreflex sensitivity in patients with obstructive sleep apnea. Am. J. Respir. Crit. Care Med..

[26] Somers V.K., Dyken M.E., Mark A.L., Abboud F.M. (1993). Sympathetic-nerve activity during sleep in normal subjects. N. Engl. J. Med..

[27] Parati G., Lombardi C., Narkiewicz K. (2007). Sleep apnea: Epidemiology, pathophysiology, and relation to cardiovascular risk. Am. J. Physiol. Regul. Integr. Comp. Physiol..

[28] Somers V.K., Mark A.L., Zavala D.C., Abboud F.M. (1989). Contrasting effects of hypoxia and hypercapnia on ventilation and sympathetic activity in humans. J. Appl. Physiol..

[29] Cintra F.D., Leite R.P., Storti L.J., Bittencourt L.A., Poyares D., Castro L., Tufic S., Paola A. (2014). Sleep Apnea and Nocturnal Cardiac Arrhythmia: A Populational Study. Arquivos Bras. Cardiol..

[30] Conway J., Boon N., Jones J.V., Sleight P. (1983). Involvement of the baroreceptor reflexes in the changes in blood pressure with sleep and mental arousal. Hypertension.

[31] Appelberg J., Nordahl G., Janson C. (2000). Lung volume and its correlation to nocturnal apnoea and desaturation. Respir. Med..

[32] Abdeyrim A., Zhang Y., Li N. (2015). Impact of obstructive sleep apnea on lung volumes and mechanical properties of the respiratory system in overweight and obese individuals. BMC Pulm. Med..

[33] Malley W.J. (2005). Clinical Blood Gases.

[34] Dempsey J. (2004). Crossing the apnoeic threshold: Causes and consequences. Exp. Physiol..

[35] Kawata N., Tatsumi K., Terada J., Tada Y., Tanabe N., Takiguchi Y., Kuriyama T. (2007). Daytime hypercapnia in obstructive sleep apnea syndrome. Chest.

[36] Temesi J., Arnal P.J., Davranche K., Bonnefoy R., Levy P., Verges S., Millet G.Y. (2013). Does central fatigue explain reduced cycling after complete sleep deprivation?. Med. Sci. Sports Exerc..

[37] Matsumoto Y., Mishima K., Satoh K., Shimizu T., Hishikawa Y. (2002). Physical activity increases the dissociation between subjective sleepiness and objective performance levels during extended wakefulness in human. Neurosci. Lett..

[38] Kamphuis J., Meerlo P., Koolhaas J.M., Lancel M. (2012). Poor sleep as a potential causal factor in aggression and violence. Sleep Med..

[39] Wåhlin Larsson B., Kadi F., Ulfberg J., Piehl Aulin K. (2008). Skeletal muscle morphology and aerobic capacity in patients with obstructive sleep apnoea syndrome. Respiration.

[40] Sauleda J., García-Palmer F.J., Tarraga S., Maimó A., Palou A., Agustí A.G. (2003). Skeletal muscle changes in patients with obstructive sleep apnoea syndrome. Respir. Med..

[41] El-Manshawi A., Killian K.J., Summers E., Jones N.L. (1986). Breathlessness during exercise with and without resistive loading. J. Appl. Physiol..

[42] Aguillard R.N., Riedel B.W., Lichstein K.L., Grieve F.G., Johnson C.T., Noe S.L. (1998). Daytime functioning in obstructive sleep apnea patients: Exercise tolerance, subjective fatigue, and sleepiness. Appl. Psychophysiol. Biofeedback.

